# South African Buffalo-Derived *Theileria parva* Is Distinct From Other Buffalo and Cattle-Derived *T. parva*

**DOI:** 10.3389/fgene.2021.666096

**Published:** 2021-06-25

**Authors:** Boitumelo B. Maboko, Kgomotso P. Sibeko-Matjila, Rian Pierneef, Wai Y. Chan, Antoinette Josemans, Ratselane D. Marumo, Sikhumbuzo Mbizeni, Abdalla A. Latif, Ben J. Mans

**Affiliations:** ^1^Agricultural Research Council, Onderstepoort Veterinary Research, Pretoria, South Africa; ^2^Department of Veterinary Tropical Diseases, University of Pretoria, Pretoria, South Africa; ^3^Agricultural Research Council, Biotechnology Platform, Pretoria, South Africa; ^4^Department of Agriculture and Animal Health, University of South Africa, Pretoria, South Africa; ^5^School of Life Sciences, University of KwaZulu Natal, Durban, South Africa; ^6^Department of Life and Consumer Sciences, University of South Africa, Pretoria, South Africa

**Keywords:** *Theileria parva*, whole genome sequencing, cattle, buffalo, genetic diversity, South Africa

## Abstract

*Theileria parva* is a protozoan parasite transmitted by the brown-eared ticks, *Rhipicephalus appendiculatus* and *Rhipicephalus zambeziensis*. Buffaloes are the parasite’s ancestral host, with cattle being the most recent host. The parasite has two transmission modes namely, cattle–cattle and buffalo–cattle transmission. Cattle–cattle *T. parva* transmission causes East Coast fever (ECF) and January disease syndromes. Buffalo to cattle transmission causes Corridor disease. Knowledge on the genetic diversity of South African *T. parva* populations will assist in determining its origin, evolution and identify any cattle–cattle transmitted strains. To achieve this, genomic DNA of blood and *in vitro* culture material infected with South African isolates (8160, 8301, 8200, 9620, 9656, 9679, Johnston, KNP2, HL3, KNP102, 9574, and 9581) were extracted and paired-end whole genome sequencing using Illumina HiSeq 2500 was performed. East and southern African sample data (Chitongo Z2, Katete B2, Kiambu Z464/C12, Mandali Z22H10, Entebbe, Nyakizu, Katumba, Buffalo LAWR, and Buffalo Z5E5) was also added for comparative purposes. Data was analyzed using BWA and SAMtools variant calling with the *T. parva* Muguga genome sequence used as a reference. Buffalo-derived strains had higher genetic diversity, with twice the number of variants compared to cattle-derived strains, confirming that buffaloes are ancestral reservoir hosts of *T. parva*. Host specific SNPs, however, could not be identified among the selected 74 gene sequences. Phylogenetically, strains tended to cluster by host with South African buffalo-derived strains clustering with buffalo-derived strains. Among the buffalo-derived strains, South African strains were genetically divergent from other buffalo-derived strains indicating possible geographic sub-structuring. Geographic sub- structuring was also observed within South Africa strains. The knowledge generated from this study indicates that to date, ECF is not circulating in buffalo from South Africa. It also shows that *T. parva* has historically been present in buffalo from South Africa before the introduction of ECF and was not introduced into buffalo during the ECF epidemic.

## Introduction

*Theileria parva* is a protozoan parasite transmitted by brown-eared ticks, *Rhipicephalus appendiculatus* and *Rhipicephalus zambeziensis* (Blouin and Stoltsz, 1989). Cape buffaloes are considered the parasite’s ancestral host, with cattle being a more recent host (Norval et al., 1991). The parasite has two transmission modes namely, cattle–cattle and buffalo–cattle transmission. Cattle–cattle *T. parva* transmission by ticks causes East Coast fever (ECF) and January disease syndromes (Theiler, 1904; Lawrence, 1979). East Coast fever is endemic in different East and southern African countries, notably Kenya, Rwanda, Tanzania, Uganda, Malawi, Mozambique, and Zambia (Lawrence et al., 1992, 2017). The name East Coast fever is derived from the historical origin of the disease in the East Coast of Africa (Theiler, 1904). South Africa had ECF outbreaks in the early 1900s that caused extensive mortalities before its eradication in the country in the 1950s (Potgieter et al., 1988; Norval et al., 1992). January disease is only reported in Zimbabwe (Lawrence, 1992; Geysen et al., 1999). These diseases cause major cattle and financial losses with January disease’s seasonality making it the milder of the two diseases due to lesser mortalities (Matson, 1967; Lawrence, 1979; Norval et al., 1992).

Buffalo–cattle transmission causes a disease known as Corridor disease. The name is derived from an outbreak reported between the corridor of the then Hluhluwe and Imfolozi nature reserves (Neitz et al., 1955). Currently, Corridor disease is endemic in certain regions of South Africa, Mpumalanga, Limpopo, and KwaZulu-Natal provinces where cattle and game share grazing land (Potgieter et al., 1988; Stoltsz, 1989; Mbizeni et al., 2013). It is very lethal to cattle to an extent that parasites rarely reach the tick-infective piroplasm stage in this host. If piroplasms are present, they are not regularly transmitted to cattle (Young et al., 1973; Uilenberg, 1999; Morrison, 2015). Barnett and Brocklesby (1966) showed that passage of buffalo-derived *T. parva* in cattle transforms the parasite to become cattle–cattle transmitted *T. parva* (Lawrence, 1992). Conversely, Potgieter et al. (1988) could not reproduce the transformation of South African buffalo-derived *T. parva* to resemble ECF strains. The latter study suggested that possible selection of cattle infective strains instead of behavioral change of the parasite may have been responsible for what was reported as “transformation.”

Recovery from *T. parva* infection can result in cattle being immune to specific parasite strains, but remain piroplasm carriers that are able to transmit the parasite to other susceptible hosts, a phenomenon known as carrier status (Young et al., 1981; Kariuki et al., 1995). The presence of carrier animals means year-round sources of parasites that can be picked up by different life stages of the vector tick, resulting in year round outbreaks. The carrier state is therefore a mode of the parasite to maintain itself in the field (Young et al., 1986; Kariuki et al., 1995; Latif et al., 2001). Naturally recovered life-long carriers of *T. parva* are common in African countries where cattle–cattle transmission is common (Kariuki et al., 1995). Asymptomatic *T. parva* positive cattle (Yusufmia et al., 2010) as well as strains with antigenic gene sequences identical to the Muguga isolate (Sibeko et al., 2010, 2011) have been reported in KwaZulu-Natal, raising concerns of the possible circulation of ECF strains in South Africa. The animals, however, lost their parasite load approximately 120 days post infection and could not transmit the parasite (Mbizeni et al., 2013).

There is genetic evidence that phenotypic differences between cattle-derived and buffalo-derived parasite strains have an underlying genetic component. A deletion in the p67 gene (allele 1) has been associated with strains of cattle origin, whereas lack of that deletion indicated buffalo origin (allele 2) (Nene et al., 1996). The observation that cattle-derived *T. parva* showed genotypes found in buffalo was first observed in South Africa (Sibeko et al., 2010) later confirmed by Mukolwe et al. (2020) and Mwamuye et al. (2020) using strains from various countries. Sibeko et al. (2010) found both alleles and additional ones in South African strains, irrespective of host origin indicating that South African populations are different from East African populations. Thus, the p67 gene could not differentiate host-derived *T. parva* in South Africa. On the other hand, 200 random genes selected by Hayashida et al. (2013) showed phylogenetic differentiation of the different host strains.

Characterization of *T. parva* has been extensively done using various loci specific molecular tools. Bishop et al. (2001) used PIM and p104 to identify different cattle-derived *T. parva* isolates, which was supported by Norling et al. (2015) using whole genome sequencing. In addition, Pelle et al. (2011) found that a less diverse population subset of *T. parva* is maintained in cattle for cattle–cattle transmission. Microsatellites have found buffalo-derived strains to be geographically diverse and the strains to be distinct from cattle-derived strains. They have also shown evidence of allele sharing among hosts in different countries (Oura et al., 2011; Elisa et al., 2014; Hemmink et al., 2018).

Next generation sequencing (NGS) has opened various avenues for research into genomic diversity. In *T. parva* for example, NGS allowed access to additional markers to characterize the parasite and discriminate ECF and Corridor disease on a genome-wide scale (Gardner et al., 2005; Hayashida et al., 2013; Norling et al., 2015). In spite of the distinction between cattle-derived and buffalo-derived strains, regions responsible for this are yet to be identified. It is thus important to identify genetic loci linked to buffalo-transmitted *T. parva* (Corridor disease) only and not cattle–cattle transmitted *T. parva* (January disease and ECF) in a region like South Africa. This may also allow the discovery of genes responsible for host adaptation.

Currently, *T. parva* whole genome sequences have been generated for eastern and southern African (ESA) strains only. While this is an important region for ECF where cattle–cattle transmission is predominant, these genomic sequences only capture genetic diversity in a subset of the geographical range of *T. parva*. To expand our understanding of the genetic diversity of *T. parva*, we have sequenced the genomes of South African strains where the primary transmission (buffalo–cattle) is different.

## Materials and Methods

### Sample History and Background

Samples used in this study were from ARC-OVR *in vitro* biobank, field collections and on-going research (Table 1).

**TABLE 1:** Source of samples used in the genomic characterization of *Theileria parva*.

**Strain**	**Accession**	**Host**	**Location/Province^*a*^**	**Origin**
8160	SAMN18117497	Bovine	Hluhluwe Nature Reserve/KZN	Game reserve buffalo (CD endemic zone)
8301	SAMN18117498	Buffalo	Welgevonden Private Game Reserve/LP	Pickup and transmission from buffalo to bovine
8200	SAMN16622817	Bovine	Ithala Game Reserve/KZN	Pickup and transmission from buffalo to bovine
9620	SAMN16622818	Bovine pastures	Pongola/KZN	Corridor disease outbreak in bovines
9656	SAMN18117499	Bovine pastures	Nyalisa/KZN	Corridor disease outbreak in bovines
9679	SAMN16622819	Bovine	Pongola/KZN Private Game Reserve	Game reserve buffalo (CD endemic zone)
Johnston	SAMN18117500	Bovine	Ladysmith/KZN	Corridor disease outbreak in bovines
KNP2	SAMN18117501	Buffalo	Kruger National Park/LP_MP	Game reserve buffalo (CD endemic zone)
HL3	SAMN18117502	Buffalo	Hluhluwe Nature Reserve/KZN	Game reserve buffalo (CD endemic zone)
KNP102	SAMN16622805	Buffalo	Kruger National Park/LP_MP	Game reserve buffalo (CD endemic zone)
9574	SAMN16622808	Bovine	Welgevonden Private Game Reserve/LP	Corridor disease outbreak in bovines
9581	SAMN18117503	Buffalo	Belvedere Game Reserve/KZN	Game reserve buffalo (CD endemic zone)

*^*a*^KZN, KwaZulu-Natal; LP, Limpopo; MP, Mpumalanga.*

#### Hluhluwe 3 (HL3)

Engorged and unengorged *Rhipicephalus appendiculatus* adults, larvae and nymphs were collected from buffaloes at Hluhluwe Game Reserve and divided into batches from which tick stabilates were made. Theilerial isolations from engorged larvae and nymphs were put in batch 3 and called isolate 3. Initially, HL3 was maintained in the laboratory by cattle–cattle passages (De Vos, 1982; Potgieter et al., 1988) and later maintained through *in vitro* culturing. The *in vitro* biobank reference for HL3 used in this study was 1/1/6/607.

#### 8160

Bovine 9433 was infected with the buffalo-derived HL3 *T. parva* strain through a blood transfusion from bovine 9266. In 2006, *Rhipicephalus zambeziensis* nymphal ticks fed on bovine 9433, picking up the parasite and transmitting it to bovine 8160. *In vitro* cultures of 8160 were subsequently initiated and used in this study. It is a third generation cattle-derived strain originating from HL3. The *in vitro* biobank reference for 8160 used in this study was 1/12/2/826.

#### 8301

*Rhipicephalus appendiculatus* ticks that had previously fed on two buffaloes from Welgevonden Private Game Reserve in 2004 were fed on bovine 9288 (Sibeko et al., 2008). *Rhipicephalus zambeziensis* ticks then picked up *T. parva* from 9288 in 2006 and transmitted it to bovine 8301. *In vitro* cultures of 8301 were subsequently initiated and used in this study. It is a second generation cattle-derived strain originating from the Welgevonden buffalo. The *in vitro* biobank reference for 8301 used in this study was 1/4/4/818.

#### 9574

*Rhipicephalus appendiculatus* ticks that had picked up *T. parva* from bovine carrier 8301 fed and transmitted the parasite to bovine 9574 at ARC-OVR quarantine facility. It is a third generation cattle-derived strain originating from the Welgevonden buffalo. Whole blood for the current work was drawn in 2017.

#### 8200

Bovine 8200 received adult *Rhipicephalus appendiculatus* collected as engorged nymphs from a buffalo in Ithala Game reserve in 2006. It had severe body reactions but recovered spontaneously without treatment to become a *T. parva* parasite carrier. *In vitro* cultures were subsequently initiated and used in this study. 8200 is considered a first generation cattle derived *T. parva* strain from buffalo strains. The *in vitro* biobank reference for 8200 used in this study was 1/12/2/844.

#### 9620 and 9656

*Rhipicephalus appendiculatus* ticks were collected from bovine pastures with a history of Corridor disease outbreaks in Pongola in 2017 and not from a game reserve as erroneously reported by Zweygarth et al. (2020). They fed on bovines 9620 and 9656, erroneously called 9596 by Zweygarth et al. (2020) at ARC-OVR quarantine facility. *In vitro* cultures were initiated upon presentation of *T. parva* clinical symptoms. 9620 and 9656 are considered first generation cattle-derived *T. parva* strains with an unknown prior host history. Biobank references 7/14/8 and 7/14/9 were allocated to 9620 and 9656, respectively.

#### 9581 and 9679

*Rhipicephalus appendiculatus* ticks collected from buffalo pastures in Pongola in 2017 and 2018, respectively, were fed on bovines 9581 and 9679 at ARC-OVR quarantine facility. *In vitro* cultures were initiated and maintained from lymph node aspirates after the bovines showed *T. parva* clinical signs. 9581 and 9679 *in vitro* cultures were subsequently initiated for the current study. 9581 and 9679 are first generation *T. parva* strains originating from buffalo pastures. Biobank references 7/14/10 and 7/18/3 were allocated to 9581 and 9679, respectively.

#### Johnston

A Corridor outbreak was suspected on a cattle farm near Pongola in 2010, based on observed clinical reactions. Lymph node biopsies were collected and *in vitro* cultures were subsequently initiated and used in this study (Latif and Mans, 2010). Johnston is considered a first generation cattle-derived *T. parva* strain with an unknown prior host history. The *in vitro* biobank reference for Johnston used in this study was 8/48/7/632.

#### KNP2

*Theileria parva* negative, laboratory reared *Rhipicephalus zambeziensis* fed on a naturally *T. parva* infected captive buffalo at the Kruger National Park (KNP) (Collins and Allsopp, 1999), which borders two provinces Mpumalanga and Limpopo (LP_MP). The ticks fed and transmitted the parasite to a bovine at ARC-OVR quarantine facility. *In vitro* cultures of KNP2 were then initiated upon presentation of *T. parva* clinical symptoms. KNP2 is a first generation cattle-derived *T. parva* strain originating from a buffalo carrier. The *in vitro* biobank reference for KNP2 used in this study was 7/14/4.

#### KNP102

Buffalo KNP102 originating from Kruger National Park in LP_MP, was donated by the South African National Parks to the University of Pretoria/Agricultural Research Council in 2004 as part of the UP/ARC BioPad consortium (Sibeko et al., 2008). It was later handed over to the ARC and has been in a tick free environment at ARC-OVR quarantine facility ever since. Whole blood for the current work was drawn in 2017. KNP102 is a zero generation buffalo-derived strain assumed to have been infected trans-placental.

Transmission experiments were done within the ARC-OVR- East Coast fever quarantine facility with ethical clearance under project 000760-Y5 and Department of Agriculture, Land reform and Rural development (DALRRD) (Section 20 12/11/1/1). *In vitro* cell cultures were established using blood and lymph aspirates from clinical animals as described with modifications (Brown, 1987). Briefly, blood and lymph aspirates were collected in heparin tubes. Lymphocytes were separated and collected using equal volume Percoll pH 8.5–9.5 (Sigma-Aldrich, United States) and washed with Dulbelcco’s phosphate buffered saline (PBS) (Sigma-Aldrich, United States). Prior to *T. parva* induced cell transformation, bovine endothelial cell lines (BA 886) (Yunker et al., 1988; Zweygarth et al., 1997) were used as feeder cells to assist in parasite multiplication. HL-1 medium was supplemented with 10% heat inactivated bovine serum or sterile fetal calf serum, 2 mM L-glutamine, 100 IU/ml penicillin, 100 μg/ml streptomycin and 2.5 mg/l amphotericin B. Cultures were incubated at 37°C. CO_2_ was only supplemented when cultures struggled to grow.

### *Theileria parva* Detection

Genomic DNA was extracted from *in vitro* cell cultures and blood material using MPLC or MP96 instruments with their respective MagNa Pure LC DNA Isolation Kit – Large Volume or MagNA Pure 96 DNA and Viral Nucleic Acid Small Volume Kit (Roche Diagnostics, Mannheim, Germany), according to manufacturer’s instructions. Eluted DNA was tested for *T. parva* using the Hybrid II assay (Pienaar et al., 2011). Molecular biology work was done in a South African National Accreditation System (SANAS) accredited (V0017) and Department of Agriculture, Land Reform and Rural development (DALRRD) (DAFF-30) approved laboratory.

### Genomic Library Preparation

Genomic library preparations for the various samples were as described by Maboko et al. (2021).

Library preparation, hybridization capture, and sequencing were conducted at ARC-Biotechnology Platform using Agilent SureSelect^*XT*^ Target Enrichment System (Agilent Technologies, Santa Clara, CA, United States) for Illumina paired-end Multiplexed Sequencing Library protocol version C1 according to the manufacturer’s instructions. Briefly, the sample enrichment process involved fragmentation of the DNA using Covaris E220 sonication (Covaris Inc., United States) set to duty factor 10, Peak Power 175 and 200 cycles per burst, resulting in a fragment size distribution of 125 bp. This was followed by purification, end-repair, addition of a 3′ -A and ligation using SureSelect adapters. Adapter-mediated PCR was done on the fragmented DNA prior to in-solution hybridization. Hybridization was conducted at 65°C for 16–24 h. Streptavidin Dynabeads (Invitrogen Carlsbad, CA, United States) were used to isolate the biotinylated baits with hybridized DNA fragments. Host DNA was washed away and captured DNA was once again amplified and indexed prior to sequencing. Library concentrations were determined using Qubit Flourometric Quantitation (Thermo Fisher Scientific, Life Technologies Holdings, Singapore) and LabChip GX Touch Nucleic Acid Analyzer (PerkinElmer Inc., United States) was used to estimate the library size. Datasets generated in the current study were submitted to GenBank under Bioproject PRJNA673089 with SRA database numbers SAMN18117497, SAMN18117498, SAMN16622817, SAMN16622818, SAMN18117499, SAMN16622819, SAMN18117500, SAMN18117501, SAMN18117502, SAMN16622805, SAMN16622808, SAMN18117503.

### Processing, Filtering and Mapping to Reference

For our genomic data to have host and regional context and for comparative consistency, single-end Illumina reads from nine East and southern Africa (ESA) *T. parva* isolates were re-analyzed (Hayashida et al., 2013) (Table 2). Fastq files were downloaded from Sequence Read Archive (SRA) of the National Center for Biotechnology Information (United States of America) and processed in the same way as our sequences except for the quality trimming and mapping which were defined as single-end reads.

**TABLE 2:** Eastern and southern African additional genomes used for the genetic diversity studies of Theileria parva*.

**Strain**	**Host**	**Year of**	**Place**	**SRA**
**name**		**isolation**	**isolated**	**accession**
Chitongo Z2	Cattle	1982	Zambia	DRR002438
Katete B2	Cattle	1989	Zambia	DRR002439
Kiambu Z464/C12	Cattle	1972	Kenya	DRR002440
Mandali Z22H10	Cattle	1985	Zambia	DRR002441
Entebbe	Cattle	1980	Uganda	DRR002442
Nyakizu	Cattle	1979	Rwanda	DRR002443
Katumba	Cattle	1981	Tanzania	DRR002444
Buffalo LAWR	Buffalo	1990	Kenya	DRR002445
Buffalo Z5E5	Buffalo	1982	Zambia	DRR002446

*Data derived from Hayashida et al. (2013).

**FIGURE 1:**
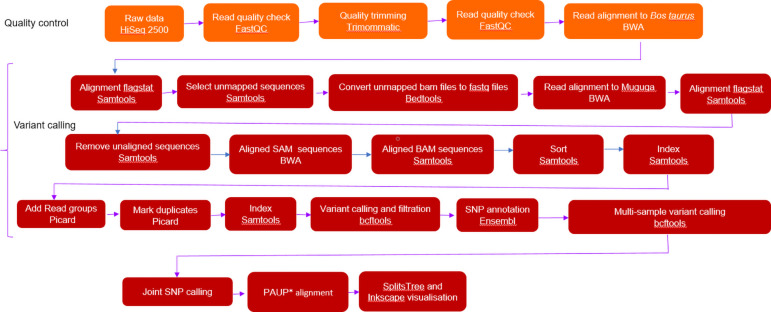
Sequential analysis workflow used for whole genome studies of *Theileria parva*. SplitsTree and Inkscape visualisation.

Data quality trimming was done by Trimmomatic (v 0.36) using a combination of SureSelect and trimmomatic-derived adapter sequences. Trimming parameters were as follows: Removed leading and trailing base quality: 4-base wide sliding window cutting when the average quality per base was below 15 (4:15). Reads below 36 bp were removed (MINLEN: 36). Sequence data quality was assessed before and after trimming using the FastQC (v 0.11.5) (Andrews, 2010). Trimmed fastq reads were first mapped to the bovine host *Bos taurus* (Hereford) genome (Accession DAAA00000000.2) using Burrows-Wheeler Aligner-Minimum Exact Match (BWA-MEM) (v 0.7.17). The default parameters used were: matching score of 1, mismatch penalty of 4, gap extension penalty of 1, gap open penalty of 6 and an unpaired read pair penalty of 9 were used except for minimum seed length (-k) of 23, which is the minimum base pair length of exact match. Bedtools (v 2.27.1) (Quinlan and Hall, 2010) was used to convert the unmapped *Bos taurus* bam files into paired fastq files to be mapped to the Muguga reference genome (Accession AAGK00000000) (Gardner et al., 2005) using BWA-MEM (v 0.7.17) also at a minimum seed length (-k) of 23. Sequences that had mapped multiple times were removed using Sequence Alignment/Map (SAMtools) (v 1.9) –F 2048 as they may represent repeat regions. PCR duplicate reads were identified using Picard MarkDuplicates (v 2.2.1) (Picard, 2019) and played no part in the subsequent downstream analysis. The general workflow for downstream analysis is indicated in Figure 1.

### Variant Calling and Phylogenetic Analysis

BCFtools mpileup was used for individual and joint variant calling. Joint variant calling was for host (buffalo and cattle), regional (South Africa and ESA) origin and the whole *T. parva* populations. Default parameters of maximum base depth of 250, minimum base quality of 13 were used. SNPs were selected from other variants using GATK SelectVariants. SNP filtration with a minimum variant quality equal and above 20 phred (QUAL), read depth (DP) equal and above 10 were called using BCFtools. VCFtools (v 0.1.15) (Danecek et al., 2011) was used to determine variant and SNP counts. Strains were analyzed as haploid organisms because the parasite is haploid in the host.

SNP-derived maximum likelihood phylogenetic tree was constructed using PAUP^∗^ with 1000 bootstraps. A reticulated tree was constructed using SplitsTree 4 (v 4.16.1) (Huson, 1998) employing the following parameters: all characters with uncorrected P parameter, neighbor-joining network at equal angles (Huson and Bryant, 2006). Inkscape v 1.0.1^1^ was used to visualise the tree.

Different strains were grouped according to host origin (buffalo and cattle). Pairwise comparison of each SNP position in individual strains was done to remove cumulative SNP counting. Shared and unique SNP positions were identified using VCFtools while SNP annotation was done using Ensembl Variant Effect Predictor (VEP) (Mclaren et al., 2016). SNP classification was based on effect of SNP on a gene, ranging from high to low. If there was no evidence of impact or it was difficult to determine whether there is an impact, it was classified as a modifier SNP. Moderate SNPs have a non-disruptive impact that might change protein effectiveness. High impact SNPs have a disruptive effect on protein function.

A total number of 74 genes of interest were selected for determining host specificity based on the following criteria (Supplementary Table 1):

a. Predicted impact of SNPs on the protein (high).
b. Subtelomeric variable secreted protein (SVSP) gene sequence associated with host–pathogen interaction (Schmuckli-Maurer et al., 2009).
c. Signal protein gene sequence involved in protein translocation (Hayashida et al., 2013).
d. Differential protein expression levels at different stages of the parasite life cycle (Tonui et al., 2018).

Our study’s strain gene sequences were obtained by searching against the Muguga sequences using BLASTN (Altschul et al., 1990). In this study, host specific SNP genes were defined as occurring in one host and not the other and are not shared among the hosts.

### Mixed Populations

To assess whether there were multiple strains in individual samples (mixed infections); a *de novo* assembly was done. Input files (fastq files) were mapped to the full-length assembled consensus sequences in order to identify the extent of intra-strain variation, which would indicate mixed infections (Mans et al., 2019). Paired-end, trimmed and host *Bos taurus* DNA filtered reads as well as ESA acquired reads were assembled using CLC Genomics Workbench (v 9.5.2) (Qiagen, Hilden, Germany) using the standard *de novo* de Bruijn graph assembly algorithm implemented in this version. The parameters used were as follows: minimum contig length: 200, mismatch cost: 2, insertion cost: 3, deletion cost: 3, length fraction: 0.5, similarity fraction: 0.8. Contig regions with low coverage were removed and the remaining ones were joined to generate final consensus sequences. Mapping parameters of reads to the assembled consensus sequences were as follows: match score: 1, mismatch cost: 2, cost of insertions and deletions: linear gap cost, insertion cost: 3, deletion cost; 3, length fraction: 0.5, similarity fraction: 0.8. Variant calling parameters were as follows: ploidy: 1, minimum coverage: 20, minimum count: 2, % minimum frequency: 35, neighborhood radius: 5, minimum central quality: 20, minimum neighborhood quality: 15.

### Population Genetic Diversity

To explore the levels of regional and host population genetic diversity, strains were separated according to host and region origin. DNASP (v 6.12.03) (Rozas et al., 2017) was used to determine the average pairwise nucleotide diversity (Pi) defined as the average number of nucleotide differences per site between pairs of DNA sequences and the fixation index (*F*_*ST*_) which measures the extent of DNA divergence between populations. *F*_*ST*_ values were interpreted as follows: low (0–0.05), intermediate (0.05–0.15), high (0.15–0.25) and very high (greater than 0.25) (Amzati et al., 2019).

## Results

### Mapping and Alignment Efficiency

Removal of contaminating host-derived reads by mapping to the *Bos taurus* genome led to unacceptable levels of incorrect mapping (loss of *T. parva* reads) when using default parameters (minimum seed length of 19). Adjustment of the BWA’s minimum seed length to 23 resulted in a reduced number of reads that incorrectly mapped to the host (*Bos taurus)*. Blood extracted strains (KNP102 and 9574) had the highest bovine sequence contamination 69% and 67%, respectively (Table 3). Removal of host-derived reads led to increased mapping percentages of reads to the parasite reference genome. Blood extracted strains also had the lowest number of mapped reads as well as average mapping coverage to the reference compared to *in vitro* culture samples. Active removal of contaminating bovine reads led to an increased mapping efficiency of approximately 20% for LAWR and Z5E5 whilst 8200 had a 50% increase. Cattle-derived strains had an improvement of 40%–66% increase with the exception of Chitongo, which had an increase of only 6%. The extent of mixed populations among South African strains was at 6% or below except for two field samples 9679 and 9581 at 86% and 77%, respectively. KNP102, kept in a tick-free environment, did not show any mixed infection (Table 4) compared to ESA strains, which had consistent extensive mixed infections except for Entebbe at 1% (Table 5).

**TABLE 3:** Mapping and sequencing statistics of different *Theileria parva* strains used in this study.

**Strain**	**Total paired reads before quality trimming**	**Total paired reads after quality trimming**	**Percentage mapped reads to *Bos taurus***	**Total paired reads used for reference mapping (Muguga)**	**Number of reads mapped to reference (Muguga)**	**Percentage mapped reads to reference (Muguga)**	**Average mapping coverage (×)**	**Genome coverage (%)**	**Total variant number**	**Total SNPs**
8200	8,938,734	8,742,536	22.06	6,669,252	6,482,174	97.19	92.366	96.61	175,224	171,748
9620	7,279,672	6,721,550	6.82	6,225,554	6,079,644	97.66	84.967	93.09	138,470	136,186
9679	7,874,554	7,062,084	8.29	6,303,390	6,111,848	96.96	85.896	88.46	66,930	65,947
KNP102	7,455,982	7,206,184	68.85	1,685,168	1,564,764	92.86	22.593	95.31	59,233	58,564
9574	8,232,602	8,028,762	67.42	2,545,358	2,419,424	95.05	34.808	93.80	120,401	118,244
8160	168,501,398	158,906,702	12.18	139,139,772	136,202,234	97.89	1917.728	97.22	196,548	192,411
KNP2	7,833,900	7,106,360	13.99	5,861,456	8,726,194	97.69	80.011	92.99	154,524	151,851
HL3	22,394,860	22,015,858	3.76	21,107,322	18,568,916	87.97	268.034	97.52	184,018	180,420
9581	8,060,570	7,870,172	11.23	6,873,992	6,651,394	96.76	94.762	97.54	94,898	93,343
9656	7,325,764	6,772,496	4.62	6,424,462	6,291,954	97.94	87.733	92.11	146,306	143,909
8301	11,114,236	10,223,658	4.87	9,587,565	9,457,338	97.85	131.847	94.44	159,577	156,709
Johnston	8,990,948	8,794,236	14.11	7,199,810	7,044,192	97.84	100.361	94.53	160,811	158,005
Chitongo	Unknown	14,405,285	10.80	12,849,208	10,179,936	79.23	43.871	94.63	23,786	23,749
Entebbe	Unknown	10,171,312	54.32	4,645,961	3,164,429	68.11	13.637	91.75	7,324	7,323
Katete	Unknown	16,558,765	62.31	6,241,639	4,528,707	72.56	19.517	94.65	12,951	12,946
Katumba	Unknown	35,406,725	84.48	5,495,080	3,727,410	67.83	16.064	94.41	10,759	10,745
Kiambu	Unknown	15,848,447	52.69	7,497,851	5,713,477	76.20	18.948	95.16	16,419	16,409
Mandali	Unknown	16,362,287	70.00	4,909,394	3,539,263	72.09	15.253	93.77	8,976	8,971
Nyakizu	Unknown	7,212,228	1.73	7,087,364	5,542,766	78.21	23.887	94.55	18,543	18,506
LAWR	Unknown	17,072,360	38.72	10,461,767	4,803,551	45.92	20.701	89.29	45,919	45,880
Z5E5	Unknown	14,821,054	39.59	8,953,444	3,968,924	44.33	17.104	90.08	36,856	36,819

**TABLE 4:** Genome features of South African *T. parva* strains.

	**8200**	**9620**	**9679**	**KNP102**	**9574**	**8160**	**KNP2**	**HL3**	**9581**	**9656**	**8301**	**Johnston**
Contig count	1,445	6,517	9,660	7,541	3946	1,299	3,935	4,939	12,482	5,806	3,676	3,410
N50 (mean length, bp)	14,679	1,885	918	1,355	3762	63,285	3,276	13,127	1,085	1,989	3,626	4,159
GC content (%)	34.24	35.03	35.43	34.71	34.83	34.28	34.82	39.10	34.89	35.07	34.69	34.63
Max. contig length	74,211	20,398	12,283	10,572	26,418	252,906	21,180	125,690	14,559	18,989	20,552	31,931
Min. contig length	200	150	151	118	188	176	184	150	118	190	126	195
Total contig length >1000 bp	7,913,602	5,668,460	3,050,776	4,081,617	6,779,720	8,260,230	6,792,895	17,845,428	4,571,833	5,586,198	7,032,359	7,099,677
Total contig length of all contigs	8,172,769	7,633,869	6,474,760	6,490,592	7,741,301	8,609,709	7,666,245	19,053,318	8,765,810	7,293,380	7,830,966	7,841,276
Total variants	1712 (1%)	8918 (6%)	57628 (86%)	0	2757 (2%)	3166 (2%)	2776 (2%)	3853 (2%)	73094 (77%)	3079 (2%)	2597 (2%)	2310 (1%)
SNPs	1,341	7,678	54,200	0	2,184	2,572	2,113	3,101	68,010	2,305	1,953	1,794

*Values in brackets represent extend of mixed variants (%) to the total number of Muguga mapped variant number in Table 3.*

**TABLE 5:** Genome features of eastern and southern African *T. parva* strains.

	**Chitongo**	**Entebbe**	**Katete**	**Katumba**	**Kiambu**	**Mandali**	**Nyakizu**	**LAWR**	**Z5E5**
Contig count	4,260	9,402	7,553	8,473	6,070	8,933	4,159	5,028	7,113
N50 (mean length, bp)	4,670	608	1,524	2,824.11	2,151	1,004	4,840	3,358	1,686
GC content (%)	34.40	37.22	34.95	34.50	34.83	35.72	34.19	34.47	34.95
Maximum contig length	42,436	5,469	21,314	17,455	21,136	14,971	48,207	43,529	18,599
Minimum contig length	199	200	200	1,367	200	200	200	199	199
Total contig length >1000 bp	6,787,839	1,355,599	4,780,159	4,380,612	5,504,375	3,182,192	6,663,018	6,129,843	4,885,893
Total contig length of all contigs	7,886,524	4,931,777	7,274,837	7,306,660	7,356,218	6,350,803	7,802,537	7,582,538	7,185,596
Total variants	7846 (1%)	7850 (33%)	8533 (66%)	9081 (84%)	8688 (53%)	9107 (101%)	8627 (47%)	6505 (14%)	8141 (22%)
SNPs	6,471	6,253	7,030	7,327	7,054	7,355	7,141	5,453	6,705

*Values in brackets represent extend of mixed variants (%) to the total number of Muguga mapped variant number in Table 3.*

South African strains had higher mapped reads percentage due to larger read length and paired-end sequencing with genome coverage at over 88%, allowing confirmation of the identity of strains as *T. parva*. The lowest average mapping coverage was at 23×, within range of good variant calling (Table 3).

The SNP alignment was 541,524 bp with 541,524 informative sites.

### Population Structure of South Africa *T. parva*

**FIGURE 2:**
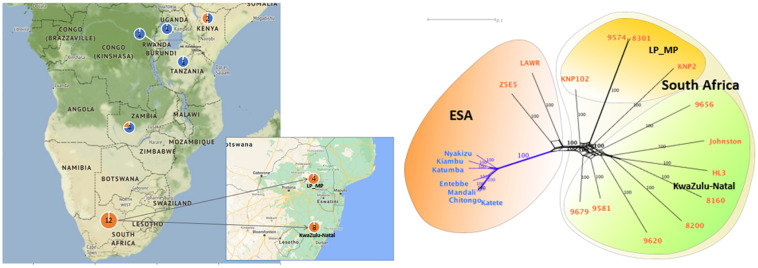
SNP phylogenetic and geographical relationships of *T. parva* strains across selected South and East African countries. Blue font represents cattle-derived strains. Orange font represents buffalo-derived strains. LP stands for Limpopo. MP stands for Mpumalanga. ESA stands for eastern and southern Africa. The phylogenetic tree was calculated by maximum likelihood analysis using PAUP*. Nodal support was assessed with 1000 bootstrap replicates. Number of nucleotide substitutions estimated at 0.01. 9574, 9656, Johnston, 8200 and 9620 were collected from a cattle host.

The South African *T. parva* strains had SNP numbers ranging from 58,684 to 192,411 compared to the Muguga reference genome (Table 3). South African strains clustered together with no differentiation between those collected from buffalo and cattle pastures (Figure 2). Most of the strains used in our study are from the KwaZulu-Natal province and these strains clustered together, with the top branch being a mixture of KwaZulu-Natal and a Limpopo/Mpumalanga strains (Figure 2). Strains isolated from bovine carrier animals (8200 and 9574), also clustered with other buffalo strains. 8160 is a Hluhluwe-Imfolozi (HL3) buffalo strain isolated from a bovine and the two strains (8160 and HL3) clustered together; similarly 8301 and 9574 that clustered together, which are second and third generations of the Welgevonden strain.

Eastern and southern African and South African strains mapped to 88.46%–97.54% of the reference genome. However, the South African strains had close to 3× more variants than ESA *T. parva* strains from the 97% of RSA and 99% of the ESA strains that were variable (segregating sites) (Table 6). Nucleotide diversity (Pi) for all strains were low across all analysis areas (geographic and host origin) but South Africa and buffalo strains tended to have higher values. Genome-wide *F*_*ST*_ values of 0.3 among South Africa and ESA supports differentiation between the two groups. All strains were distinct to an extent that haplotypes could not be determined.

**TABLE 6:** Genetic diversity of all joint *T. parva* strains.

	**Regions**		**Host**	
	**South Africa**	**Eastern and Southern Africa**	**Buffalo**	**Cattle**
Sample number	12	9	14	7
Total variant number	501,479	188,999	536,603	91,849
SNP number	485,520	187,620	520,362	91,115
Segregating/variable SNP sites	485,520	187,620	520,362	91,115
Nucleotide diversity (Pi)	0.008275	0.003034	0.005959	0.001640
Tajima’s *D*	−0.02274	−0.36771	−0.16821	0.3507380
*F*_*ST*_	0.30334		0.41099	
				

### Host Differentiation

Buffalo-derived strains (mostly from South Africa) had 6× more SNPs than the cattle-derived (Table 5) with different strains clustering according to host origin. ESA buffalo-derived strains clustered with South African buffalo-derived strains (Figure 2).

**FIGURE 3:**
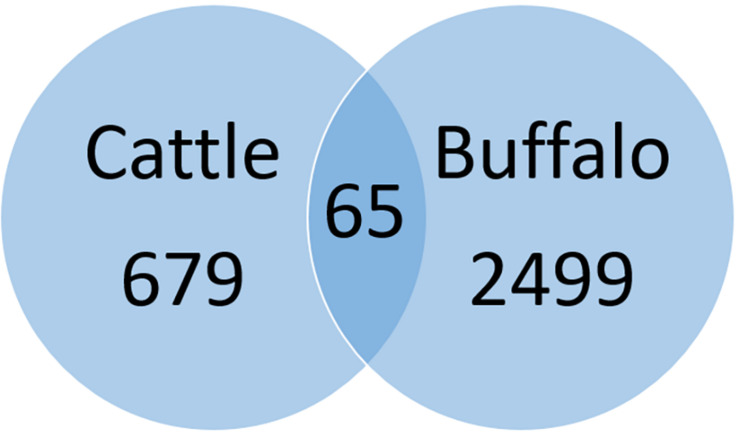
Pairwise comparison of cattle and buffalo-derived SNPs. Sixty-five SNPs were shared between cattle and buffalo strains whereas 679 and 2499 SNPs were specific to cattle and buffalo, respectively.

A total of 679 SNPs were specific to cattle, 65 SNPs were shared by the two hosts and 2499 were specific to buffaloes (Figure 3). These values include strains with mixed infections. Only two high impact SNPs were found among all identified SNPs, poly (A) polymerase and a hypothetical protein (TP02_0956), both from buffalo-derived *T. parva*. However, gene and protein multiple sequence alignments did not support the high impact classification due to limited variation between cattle- and buffalo-derived sequences.

Genome-wide *F*_*ST*_ values of 0.4 among cattle and buffalo supports differentiation between the two groups, further supported by the high nucleotide diversity observed in buffaloes (Table 6).

## Discussion

In the present study, we describe the use of whole genome sequencing for identification of genetic variants in the genomes of South African and ESA buffalo-derived *T. parva* strains sequenced by next generation sequencing. Variant calling was also done for the first time from blood extracted *T. parva* samples. This allowed a multi-locus phylogenetic analysis to determine phylogenetic relationships with greater confidence.

The study indicated that even with bait capture, host-derived DNA remains a potential contamination problem. Seed length optimization was important in getting rid of host DNA. Failure to do so leads to a high number of reads mapping to the host, leading to wrong interpretation of the purification method. Robinson et al. (2017) also found that seed length adjustment worked well in cases where parasite DNA concentration is low compared to host’s, as was the case for *Plasmodium*.

Although sample numbers were low, high SNP numbers allowed for meaningful analysis of data to determine *T. parva* diversity across host and geographical area. Available data so far supports the arguments that buffalo-derived *T. parva* is distinct from cattle-derived strains (Oura et al., 2011; Elisa et al., 2014) with cattle strains being less diverse, subset of the buffalo strains (Oura et al., 2003; Pelle et al., 2011). A similar study done by Palmateer et al. (2020), also using bait capture found buffalo-derived *T. parva* to be highly divergent from cattle-derived *T. parva*. While this latter genome would be of interest to use as an alternative reference genome to the Muguga genome, it still needs refinement with regard to assignment of contigs to the correct homologous chromosomes of the Muguga genome. Whole genome alignment indicated that large regions of various contigs do not align to the Muguga reference genome (results not shown). This suggest significant differences between the cattle- and buffalo-derived *T. parva* genomes, or that the current draft genome is not yet at reference genome status.

The clustering of all buffalo-derived strains regardless of geographic origin suggests that they have a common ancestor (Figure 2). The Cape buffalo evolved from an ancestor similar to the western African buffalo (*Syncerus brachyceros*) and is distributed over eastern and southern Africa (Smitz et al., 2013). It is believed that the Cape buffalo has always been infected with *T. parva* (Young et al., 1978; Mans, 2020), which explains the clustering of buffalo-derived *T. parva* while being distinct due to lack of gene flow between the two regionally distinct strains.

Nucleotide diversity and *F*_*ST*_ values were skewed toward higher numbers in buffalo-derived *T. parva* as indicated by South African strains that were exclusively buffalo-derived. Similarly, clustering of cattle-derived ECF strains suggest that they also share a common ancestor that may have resulted from a single origin from the more diverse East African buffalo-derived stock (Obara et al., 2015; Nene and Morrison, 2016). Previous suggestions that *T. parva* originated in East Africa (Norval et al., 1991) may need to be reassessed, since the high diversity observed in southern African strains would suggest that *T. parva* rather originated in this latter region. However, more data from ESA buffalo- and cattle-derived *T. parva* is necessary to confirm the higher diversity observed in South Africa that would support this hypothesis.

Buffalo-derived strains had more substitutions (longer branch lengths) suggesting that cattle-derived strains separated from buffalo-derived strains at a later stage. 8200, 9581, 9620, 9679 and 9656 are first generation cattle-associated strains of buffalo origin that show no sign of population selection. Similarly, 8160 is a second-generation buffalo-derived strain with at least two cattle-dependent bottlenecks (transmission from buffalo HL3 to bovine 9433 to bovine 8160), but still clusters with HL3 with no sign of population selection. Correspondingly, 9574, a third generation buffalo-derived strain that was tick passaged through three cattle-dependent bottlenecks (transmission from buffalo Welgevonden to bovine 9288 to bovine 8301 to bovine 9574), still clusters with 8301 (Figure 2). Parasites that were derived from cattle in their latest passage have not transformed or been strain selected for cattle–cattle transmission. In the latter case, it is expected that they would have clustered with other cattle *T. parva* strains (Figure 2). In South Africa, the seasonality observed for the various tick life stages (Mbizeni et al., 2013), may decrease the likelihood of strains going through several cattle passages for transformation like in Barnett and Brocklesby (1966). Selection of cattle transmissible strains as suggested by Potgieter et al. (1988) is also unlikely because buffaloes and cattle are not permitted to come into contact (Department of Agriculture, 1984). Therefore, these parasites are cattle associated strains of buffalo origin that are infective to cattle. Buffalo movement is restricted in South Africa and therefore, *T. parva* positive buffaloes can only be moved to *T. parva* endemic areas and thus clustering of KwaZulu-Natal and Limpopo/Mpumalanga strains is not surprising.

Mixed populations were observed in two field strains (9679 and 9581) which had over 70% intra-strain variation (Table 4). These field strains had recently been introduced into *in vitro* culturing and strain selection may have not occurred as reported for cell-cultured *T. parva* schizonts (Kasai et al., 2016).

*Theileria* subtelomeric variable proteins and signal proteins expressed in the schizont phase are believed to be involved in host cell manipulation (Shiels et al., 2006; Schmuckli-Maurer et al., 2009) and thus may be involved in host specificity. The inability to find specific regions or genes responsible for host specificity from the selected genes may indicate that other unidentified and/or unselected gene regions are responsible. It is therefore necessary to explore additional genes including PIN1, a gene previously unknown and identified during the recent re-annotation of *T. parva* (Tretina et al., 2020) as well as the other 127 previously unknown genes. It is also possible that buffalo diversity is so great that no unique SNP markers shared by all buffalo could be found. This study also did not find the p67 diversity reported by Mukolwe et al. (2020). A likely reason for this is that their study used blood extracted field samples, which may have had multiple *T. parva* strains whereas the current work used clonal *in vitro* samples.

In South Africa, buffaloes are only allowed to move to endemic regions, this might lead to increased parasite diversity as a result of sexual reproduction of the parasite in ticks, which will explain the lack of haplotypes and all distinct strains (Mehlhorn and Shein, 1984). Differentiation of South African strains from ESA strains may be as a result of geographic separation. Genetic drift is most likely due to the strict control measures in South Africa regarding buffalo movement and wildlife-livestock interaction as well as theileriosis control measures. This lack of interaction prevents the parasite from being selected for cattle–cattle transmission and a possible reason why South Africa does not have cattle-adapted *T. parva* populations that would allow cattle–cattle transmission that would resemble ECF.

Based on heavy mortalities of domestic livestock during the ECF epidemic in South Africa and the discovery of Corridor disease after eradication of ECF from South Africa, it was proposed that *T. parva* did not circulate in African buffalo in southern Africa before the introduction of ECF, but was transmitted to and established in buffalo during the ECF epidemic (Norval et al., 1991, 1992). Recently it was suggested that infected cattle introduced into South Africa did not carry buffalo-derived *T. parva*, but that extensive genetic diversity of buffalo-derived *T. parva* from South Africa suggest that this parasite was present in South Africa before introduction of ECF (Mans, 2020; Morrison et al., 2020). For the alternative scenario where cattle-derived *T. parva* established in buffalo during the ECF epidemic, a low genetically diverse population would have been expected, since at least two genetic bottlenecks occurred for cattle-derived *T. parva* introduced into South Africa. The first bottleneck occurred as adaptation of buffalo-derived *T. parva* to cattle in East Africa that resulted in the genetically restricted low diversity populations currently observed for cattle-derived *T. parva* throughout East and southern Africa. The second bottleneck occurred when cattle-derived *T. parva* was introduced into South Africa from a single or restricted number of *T. parva* populations (Norval et al., 1991). However, the current study provides concrete evidence that buffalo-derived *T. parva* from South Africa shows very high genetic diversity and is distinct from ECF strains. This genetic diversity would suggest an ancient association of *T. parva* with African buffalo from southern Africa.

## Conclusion

Our data indicate that *T. parva* strains circulating in South Africa are buffalo derived, associated with Corridor disease and that there are no ECF circulating South African strains discovered to date in buffalo. Further work still needs to be done to identify regions responsible for host specificity.

## Data Availability Statement

The datasets presented in this study can be found in online repositories. The names of the repository/repositories and accession number(s) can be found below: https://dataview.ncbi.nlm.nih.gov/object/PRJNA673089?reviewer=42193fcf2rb2g9jk5k9o10u7rj, SAMN18117497, SAMN18117498, SAMN16622817, SAMN16622818, SAMN18117499, SAMN16622819, SAMN18117500, SAMN18117501, SAMN18117502, SAMN16622805, SAMN16622808, and SAMN18117503.

## Ethics Statement

Ethics approval was obtained from the ARC-OVR (project 000760-Y5) and University of Pretoria (V033-17). Written permission from national authorities was obtained.

## Author Contributions

BBM developed the method, designed the experiments, performed the data analysis, and wrote the manuscript. KS-M co-supervised, performed the data analysis and critically reviewed the manuscript. RP assisted in data analysis and critically reviewed the manuscript. AJ assisted in experiments and critically reviewed the manuscript. RM and SM assisted in all animal related experiments and critically reviewed the manuscript. AL provided intellectual assistance and critically reviewed the manuscript. BJM supervised, conceived the idea, and critically reviewed the manuscript. All authors contributed to the article and approved the submitted version.

## Conflict of Interest

The authors declare that the research was conducted in the absence of any commercial or financial relationships that could be construed as a potential conflict of interest.

## Footnotes

1 Harrington, B. et al (2004-2005). Inkscape. http://www.inkscape.org/.

## Supplementary Material

Supplementary Table 1 Genes of interest studied to determine host specificity.
