# Supplementary material for: South African Buffalo-Derived Theileria parva Is Distinct From Other Buffalo and Cattle-Derived T. parva
Source: Front Genet. 2021 Jun 25;12:666096. doi: 10.3389/fgene.2021.666096 (PMC8269612; doi:10.3389/fgene.2021.666096)
Supplement: Supplementary Table 1 — Genes of interest studied to determine host specificity. [file Table_1.DOCX]

# **SUPPLEMENTARY MATERIAL**

## **Additional file 1:** Table S1. Genes of interest studied to determine host specificity

## **Table S1**

## Genes of interest studied to determine host specificity

| **Gene** | **Gene name** | **Consequence** | **Function** |
| --- | --- | --- | --- |
| TP01_0037 | Poly(A) polymerase | Splice donor variant |  |
| TP01_0144 | HP | Downstream gene variant | Signal protein |
| TP01_0291 | HP | Upstream and downstream gene variants | Signal protein |
| TP01_0293** | 60S ribosomal protein L38 | Downstream gene variant |  |
| TP01_0380 | HP | Downstream gene variant | Signal protein |
| TP01_0541** | HP | Missense, downstream, synonymous gene variant |  |
| TP01_0560** | 60S ribosomal protein L3 | Upstream gene variant |  |
| TP01_0610 | HP | Upstream gene variant | Signal protein |
| TP01_0613* | SuAT_1_-like protein | Upstream and downstream gene variants |  |
| TP01_0621 | HP | Missense and downstream gene variant | Signal protein |
| TP01_0673** | HP:H/ACA ribonucleoprotein complex, subunit Nop10 | Downstream gene variant |  |
| TP01_0675** | 60S ribosomal protein L14 | Downstream gene variant |  |
| TP01_0726** | Elongation factor 1 alpha | Upstream gene variant |  |
| TP01_0923^#^ | DEAD-box RNA helicase | Upstream gene variant |  |
| TP01_0914 | HP | Missense gene variant | Signal protein |
| TP01_0934* | HSP90 family | Upstream gene variant |  |
| TP01_0955 | HP | Downstream gene variant | Signal protein |
| TP01_1182** | L-lactate dehydrogenase | Downstream, missense and synonymous gene variant |  |
| TP01_1225 | HP: Conserved *Theileria*-specific sub-telomeric protein, SVSP family, putative | Downstream gene variant | SVSP |
| TP01_1226 | HP: Conserved *Theileria*-specific sub-telomeric protein, SVSP family, putative | Missense and downstream gene variants | SVSP |
| TP01_1227 | HP | Upstream and missense gene variants | Signal & SVSP protein |
| TP02_0007 | HP | Upstream gene variant | SVSP |
| TP02_0008 | HP | Upstream gene variant | SVSP |
| TP02_0010 | HP | Upstream and missense gene variants | Signal & SVSP protein |
| TP02_0011^#^ | HP | Missense gene variant | SVSP |
| TP02_0018 | HP | Upstream gene variant | Signal protein |
| TP02_0036** | HP: Protein yippee-like | Downstream gene variant |  |
| TP02_0370** | 60S acidic ribosomal protein P2 | Upstream gene variant |  |
| TP02_0551 | 23kDa piroplasm surface protein | Upstream and downstream gene variant | Signal protein |
| TP02_0575 | HP | Synonymous gene variant | Signal protein |
| TP02_0600** | Proliferating cell nuclear antigen | Upstream gene variant |  |
| TP02_0875 | HP | Missense gene variant | Signal protein |
| TP02_0956* | HP: *Theileria*-specific sub-telomeric protein, SVSP family, putative | Stop codon lost |  |
| TP03_0039 | HP | Upstream and missense gene variant | Signal protein |
| TP03_0152** | Histone H3 | Downstream gene variant |  |
| TP03_0193** | HP | Upstream gene variant |  |
| TP03_0217 | HP | Downstream gene variant | Signal protein |
| TP03_0282* | Cysteine protease precursor, tacP | Upstream, downstream and synonymous gene variants |  |
| TP03_0283* | Cysteine protenase precursor, tacP | Upstream, downstream and synonymous gene variants |  |
| TP03_0284* | Cysteine protenase precursor, tacP | Upstream and downstream gene variant |  |
| TP03_0298 | HP | Upstream gene variant | Signal protein |
| TP03_0299** | HP | Upstream gene variant |  |
| TP03_0313** | HP | Missense gene variant |  |
| TP03_0488** | 60S ribosomal protein L34a | Downstream gene variant |  |
| TP03_0498 | HP | Upstream, downstream and missense gene variants | Signal protein |
| TP03_0755** | 60S ribosomal protein L15 | Downstream gene variant |  |
| TP03_0810 | Hypothetical Protein | Upstream gene variant | Signal protein |
| TP03_0883 | HP: Protein of unknown function DUF529 | Downstream gene variant | SVSP |
| TP03_0884 | HP: Conserved *Theileria*-specific sub-telomeric protein, SVSP family, putative | Upstream and downstream gene variant | SVSP |
| TP03_0869* | HP: Conserved *Theileria*-specific sub-telomeric protein, SVSP family | Upstream gene variant | SVSP |
| TP03_0885 | HP: Protein of unknown function DUF529 | Downstream gene variant | SVSP |
| TP03_0886 | HP: Conserved *Theileria*-specific sub-telomeric protein, SVSP family | Missense gene variant | Signal protein |
| TP03_0887 | HP: Protein of unknown function DUF529 | Upstream and downstream gene variant | SVSP |
| TP03_0888 | HP | Upstream gene variant | SVSP |
| TP03_0889 | HP | Upstream and downstream gene variant | SVSP |
| TP04_0010# | HP: Protein of unknown function DUF529 | Upstream gene variant | SVSP |
| TP04_0011 | HP: Protein of unknown function DUF529 | Upstream gene variant | SVSP |
| TP04_0012 | HP: Protein of unknown function DUF529 | Upstream gene variant | Signal protein |
| TP04_0013 | HP: *Theileria*-specific sub-telomeric protein, SVSP family, putative | Upstream and missense gene variant | Signal & SVSP protein |
| TP04_0014 | HP: Protein of unknown function DUF529 | Upstream, downstream, missense and synonymous gene variants | SVSP |
| TP04_0015 | HP: Protein of unknown function DUF529 | Upstream, downstream, missense, synonymous gene variants | SVSP |
| TP04_0016 | HP: Protein of unknown function DUF529 | Upstream, downstream and synonymous | SVSP |
| TP04_0018 | HP: *Theileria*-specific sub-telomeric protein, SVSP family, putative | Upstream, downstream, missense, synonymous gene variants | SVSP |
| TP04_0019 | HP*: Theileria*-specific sub-telomeric protein, SVSP family, putative | Upstream, downstream, missense, synonymous gene variants | SVSP |
| TP04_0104 | HP: Protein of unknown function DUF529 | Upstream gene variant | SVSP |
| TP04_0142** | Protein translation initiation factor SUI1 | Upstream and downstream gene variants |  |
| TP04_0275* | HP | Downstream, missense and synonymous gene variant |  |
| TP04_0404** | Histone H2B-III | Downstream gene variant |  |
| TP04_0411 | HP | Downstream gene variant | Signal |
| TP04_0700** | Enolase | Upstream gene variant |  |
| TP04_0844** | 40S ribosomal protein S7 | Upstream gene variant |  |
| TP04_0919 | HP | Downstream and missense gene variant | Signal protein |
| TP04_0920 | HP | Upstream gene variant | Signal & SVSP protein |
| TP04_0921 | HP | Upstream gene variant | Signal protein |

*HP: Hypothetical protein. SVSP: Subtelomeric variable secreted protein gene*

*^#^Gene variant unique to cattle*

** Gene variant unique to buffalo*

***High expression*
